# Should CSF Biomarkers Support a Routine Analysis for Early Diagnosis of Alzheimer's Disease?

**DOI:** 10.4061/2010/293587

**Published:** 2010-11-08

**Authors:** Lucilla Parnetti, Jens Wiltfang, Kaj Blennow

**Affiliations:** ^1^Centre for Memory Disturbances, Section of Neurology, University of Perugia, 06100 Perugia, Italy; ^2^Kliniken und Institut der Universität Duisburg-Essen, Germany; ^3^Clinical Neurochemistry Laboratory, The Sahlgrenska Academy at Göteborg University, Mölndal, 40530 Gothenburg, Sweden

In view of the availability of drugs that may slow or even halt the progression of Alzheimer's disease (AD), the possibility to routinely discriminate individuals carrying incipient AD is utmost important. The CSF biomarkers beta-amyloid and tau are increasingly used in several countries in Europe for their high diagnostic accuracy in detecting early or even prodromal AD. Up till now, thousands of subjects have been studied either in research or routine setting, giving sensitivity and specificity values invariably above 80%. Despite these clinically relevant results, CSF analysis is still considered only a supportive exam in AD diagnostic work-up. We think there is a great need to improve the medical awareness about the importance to perform lumbar puncture as a routine procedure in the diagnostic assessment of cognitive deterioration. 

In line with this belief, the first paper of this special issue addresses the usefulness of cerebrospinal fluid analysis for diagnostic definition of cognitive deficits, and the second one further focuses on the ethical issues related to early diagnosis of Alzheimer's disease. As a corollary, the third paper reports some reflections about the actual need in clinical practice of guidelines for diagnosis and management of incipient AD. 

The fourth paper illustrates what is known about CSF biomarkers for diagnosing AD, and the fifth paper gives a different perspective according to the Canadian guidelines for dementia diagnosis. An up-dated and comprehensive view of crucial issues emerged from large multicentre studies (which have clearly highlighted the inter-centre variability of these determinations) on CSF biomarkers for AD diagnosis is reported in the sixth paper. Accordingly, the seventh paper nicely describes the efforts done for standardization of assay procedures and the eighth paper further goes into details about the many factors inducing variation of amyloid beta concentration, which heavily hampers, at present, a good inter-centre reliability. Besides the state of art about what is known on classical CSF biomarkers for early diagnosis in routine clinical practice, there also are impressive international research initiatives aimed at identifying new biomarkers for neurodegenerative diseases. This issue is thoroughly addressed by the ninth paper, which gives an overview of the EU-funded consortium cNEUPRO. 

The issue of the role of CSF biomarkers in non-AD dementias is also partly addressed in this special issue. An overview on CSF biomarkers in dementia with Lewy bodies (DLB) is given in the tenth paper, and in the eleventh paper the discriminative power of classical and putative CSF biomarkers for differentiating AD, DLB, and Parkinson's disease with dementia (PDD) is accurately described; finally, the usefulness of classical CSF biomarkers in characterizing CADASIL, a genetic model of subcortical vascular dementia, is also reported.

*Lucilla Parnetti* *Lucilla Parnetti* *Jens Wiltfang* *Jens Wiltfang* *Kaj Blennow* *Kaj Blennow*

##  Tuula Pirttilä In Memoriam

Professor Tuula Pirttilä from University of Eastern Finland, Kuopio, Finland, passed away on March 24, 2010. She was fighting against cancer for almost five years. Despite of her disease, she was constantly working as clinical doctor, scientist, and teacher. 

Tuula Pirttilä was born in Kuopio. She studied Medicine at the University of Tampere and got her M.D. in 1983. She completed her residency in neurology in 1990 and got the degree of PhD three years later. After that, she moved to New York, USA and as post doc she carried out doing studies on biomarkers of Alzheimer's disease. She was nominated as docent in neurology at the University of Tampere in 1995. She then returned to Kuopio as associate professor of Neurology in 1997 and was nominated full professor of neurology in 2005. 

People who have been working with her, remember her as an excellent clinician, teacher and scientist. Ethical issues were very important for her. She served many years as a chairperson of the Ethical Committee of Kuopio University Hospital. Tuula was also an active performer in patient organisations and in Finnish Alzheimer Research Society. Among many other things she was also chairperson in the working group defining national and European guidelines for diagnosis of Alzheimer disease. Her contribution to the science was mainly focused on biomarkers. She could use excellent material collected in Kuopio Finland including plasma, serum, CSF samples from patients with neurodegenerative diseases, especially Alzheimer's disease. Her group set up a service laboratory at the University of Eastern Finland that is currently serving the whole country. Her contribution to the EU funded cNEUPRO Consortium has been decisive for the success of the initiative, and she gave her enthusiastic contribution until she could.

Tuula showed unbelievable talent and wisdom. She will be remembered as a very warm and ethusiastic person, always available to listen and engage, and her smiles and laughs were contagious.

All of us who had the privilege to work with her are missing a great person, scientist, teacher and clinician. 

*Hilkka Soininen* *Hilkka Soininen* *Lucilla Parnetti* *Lucilla Parnetti* *Jens Wiltfang* *Jens Wiltfang* *Kaj Blennow* *Kaj Blennow*

